# NorthPop: a prospective population-based birth cohort study

**DOI:** 10.1186/s12889-025-23561-y

**Published:** 2025-06-26

**Authors:** Christina E. West, Pernilla Lif Holgerson, Anna Chmielewska, Richard Lundberg-Ulfsdotter, Carina Lagerqvist, Elisabeth Stoltz Sjöström, Katharina Wulff, Olof Sandström, Ingrid Mogren, Sven Arne Silfverdal, Magnus Domellöf

**Affiliations:** 1 Department of Clinical Sciences, Pediatrics, Umeå University, 901 85 Umeå, Sweden; 2 Department of Odontology, Pedodontics, Umeå University, Umeå, Sweden; 3 Department of Food, Nutrition and Culinary Science, Umeå University, Umeå, Sweden; 4 Department of Molecular Biology, Umeå University, Umeå, Sweden; 5 Department of Clinical Sciences, Obstetrics and Gynecology, Umeå University, Umeå, Sweden

**Keywords:** Children, Developmental origins, Environment, Epidemiology, Non-communicable diseases, Nutrition, Obstetrics, Programming, Risk factors

## Abstract

**Background:**

Non-communicable diseases (NCDs) are a global health issue, posing a substantial burden on the individual, community, and public health. The risk of developing NCDs is influenced by a complex interplay between genetic, epigenetic, and environmental factors.

**Methods:**

The NorthPop Birth Cohort Study (NorthPop) constitutes an infrastructure enabling cutting-edge research on the foundational pathways to NCDs in childhood, including allergic diseases and asthma, overweight/obesity, cognitive and neurodevelopmental dysfunction, gastrointestinal disorders, and caries. NorthPop aims at recruiting 10,000 families. Pregnant women and their partners residing in Västerbotten County, Sweden are eligible. Recruitment started in 2016 and is anticipated to end in 2025. Extensive data on parental, fetal and child health outcomes, lifestyle, diet, and environmental exposures are prospectively collected using web-based questionnaires in pregnancy and childhood until the children turn 7 years old. Urine samples are collected from the pregnant woman at gestational age 14–24 weeks. Blood samples are collected at gestational age 28 weeks. Placenta and cord blood are collected at birth. A breast milk sample is collected 1 month postpartum. Blood samples from the children are collected at 18 months and 7 years of age. Oral swabs and fecal samples are collected from the children within 48 h of birth, at 1, 9 and 18 months, 3 and 7 years of age. At age 7 years, children are invited to a follow-up visit, including measurements of weight, height, blood pressure, pulse, hand grip strength, working memory, skin prick test and saliva sampling. Additional measurements, such as sleep–wake and light exposure, and additional biological samples are collected in sub-cohorts. Permission for linkage to medical records and national registers e.g., the Swedish Pregnancy Register, the National Patient Register, the Longitudinal Integration Database for Health insurance and Labor market studies and the Swedish Prescribed Drug Register has been granted.

**Discussion:**

Our multidisciplinary approach allows us to study how early life exposures, as well as parental health and lifestyle, influence future health in the offspring. Our results are anticipated to contribute to the understanding of disease risk and may inform future strategies aimed at risk reduction, highly significant for public health.

**Trial registration:**

Retrospectively registered at Researchweb 11 November 2024 (project number 279272).

## Background

The first 1,000 days of life are critical in programming the health and well-being of an individual. This is originally stemming from epidemiological studies reporting an association between low birth weight and death from ischemic heart disease in adults. The fetal origin’s hypothesis theorizes that undernutrition during gestation is an early origin of metabolic and cardiac diseases as a result of fetal programming [1]. This programming permanently shapes the body’s structure, physiology, and metabolism, thereby contributing to disease risk in adulthood. The concept has been further expanded to include other early life environmental exposures and other diseases. Notably, the Developmental Origins of Health and Disease (DOHaD) paradigm emphasizes the role of both prenatal and postnatal environmental exposures on disease risk in the immediate and long-term perspective [2]. Environmental and lifestyle exposures during early life stages are associated with risks of several major public health problems, including cardiometabolic disorders, obesity, respiratory disorders and impaired mental health.

Pregnancy is a key determinant of future health for both the mother and child. Population-based data on maternal and fetal/child health outcomes are essential in surveying the role of pregnancy and perinatal factors for the prevention, management, and improvement of health outcomes [3–5]. National Medical Birth Registers including population-based data have been invaluable for monitoring maternal, fetal and child health [3–6]. Maternal lifestyle, environmental exposures, intercurrent diseases and complications during pregnancy, may affect the fetal environment and cause changes in fetal growth, development, metabolic and immune programming, with long-term negative effects on offspring health [7]. There is now emerging evidence that not only maternal but also paternal preconception health influences the offspring’s health, possibly via epigenetic mechanisms [8, 9].

After birth, infants are exposed to microbes, diet and environmental substances involved in educating immune functions [10]. Colonization of the gut and other mucosal surfaces is critical for the development of the immune system and parallels the maturation of both the innate and adaptive immune systems [11–14]. In early life, the infant is vulnerable to infections, and has protection by maternally transferred antibodies, and antimicrobial components in breast milk [15]. After infancy, the toddler is exposed to a wide range of microbes, sometimes causing invasive disease. The societal burden of infectious diseases in childhood is heavy [16]. We have knowledge about some risk factors, and prevention via vaccinations is effective. Nutrition in early life also plays an important role [17–20] but the knowledge about all defense mechanisms against infections in childhood is still limited.

Over the past few decades, there has been, and still is, an increase in NCDs affecting the current generation of children. This increase is likely due to complex environmental, lifestyle and dietary changes as well as progressive urbanization and reduced biodiversity [21]. Early-onset NCDs include allergic diseases and asthma, overweight and obesity with its’ cardiometabolic consequences, neurodevelopmental and cognitive disorders, and mental ill-health. The prevalence of allergic diseases and asthma has increased globally [22], now afflicting a third of young adolescents in Sweden [23]. In a retrospective study, we found increasing incidence trends of emergency food allergy reactions and food-induced anaphylaxis in children and adolescents in our region [24] which is consistent with reports from several countries across the globe [25]. Still, national population-based data on food allergy evaluated by gold standard oral food challenges are lacking in many parts of the world, which is troublesome since self-reported food allergy will overestimate the true prevalence [26, 27]. In Sweden, there is a paucity of large population-based studies that have examined food allergy using objective methods e.g., oral food challenges in early childhood. Over the last decades there has also been a world-wide increase in the prevalence of celiac disease suggesting an association with modern lifestyle and dietary habits [28–30]. Celiac disease is an autoimmune enteropathy triggered by dietary gluten in wheat, rye, and barley in genetically susceptible individuals. The enteropathy results in gastrointestinal symptoms, malnutrition, ill-health, and poor quality of life and can only be treated with a diet free from gluten [30]. Since around 1 to 3% in different populations are affected, identifying possible strategies for primary prevention of celiac disease is needed [30]. Notably, there is some evidence that a higher amount of gluten introduced at weaning and/or thereafter may increase the risk of celiac disease, but the evidence is insufficient to determine the amount of gluten or types of gluten-containing foods associated with the increased risk [31].

Feeding difficulties in children are common. Every fourth otherwise healthy toddler may present with food refusal, picky (fussy) eating or selective eating of different severity [32]. These difficulties may in turn affect growth, development and cause parental stress and feeling of incompetence [33, 34]. A smaller proportion of these children suffer from pediatric feeding disorder (PFD) i.e., an inability to consume an age-appropriate diet associated with medical, nutritional, feeding skills dysfunction and/or psychosocial dysfunction [35]. There is a lack of data on the prevalence of PFD from population-based samples [36]. Data on prevalence and prognosis of avoidant restrictive feeding disorder (ARFID) in children is also limited [37]. Some eating behaviors have also been associated with the risk of overweight and obesity [38]. The prevalence of childhood overweight/obesity remains very high worldwide [39] and in Sweden about 20% of 6 to 9-year-old children are overweight or obese [40]. Early childhood diet (including breastfeeding), growth, parent–child-interaction, sleep pattern, physical activity and development of the gut microbiome are some of the relevant factors that have been suggested to impact the development of childhood overweight and obesity [41]. A better understanding of these and other risk factors will help us to develop effective preventive interventions [42].

In addition, mental health problems, including cognitive and neurodevelopmental disorders, affect approximately 20% of children in high-income countries. The childhood prevalence is 7–8% for Attention Deficit Hyperactivity Disorder (ADHD), ~ 2% for autism spectrum disorder, 5–15% for specific learning disorders and 2–3% for global developmental delay [43]. In Sweden, there is a worrying upward trend in the prevalence of mental health problems in children [44]. The brain is the fastest growing organ from the third trimester of pregnancy up to 3 years of life, when the brain has reached 85% of the adult size. Early life exposures will affect neurodevelopment; one example is nutrition, which is essential for normal brain development during the very sensitive stages of neuronal proliferation, migration, myelination, and synapse formation. We have previously shown that early interventions effectively can improve later neurodevelopment in Swedish children [45–48]. Novel exposures which may influence neurodevelopment include digital devices, screen time and social media, all of which will be possible to study in the NorthPop Birth Cohort Study (NorthPop).

The oral microbiota is under development up to 5 years of age and beyond, with fluctuations likely reflecting age-related environmental influences [49]. There is also emerging evidence that the crosstalk between the oral microbiota and the host can impact the risk of early onset NCDs [50]. In many parts of the world, children's oral and dental health require major efforts, mainly due to the multifactorial caries disease [51]. The oral microbiota and children's dietary patterns are primarily causing caries disease, but other external and internal factors contribute [52]. In Sweden, decade-long, large investments in preventive measures in dentistry led to low caries rates, but unfortunately the numbers have increased in recent years, and now nearly every fifth child is afflicted [53].

Early-onset NCDs, such as allergy, asthma, and some autoimmune diseases, and later-onset NCDs, including cardiovascular disease and associated metabolic disease, as well as mental disorders, seem to have environmental risk factors and genetic risk variants in common [54, 55]. Within a population-based longitudinal cohort study, there are numerous possibilities to study how both the environment and genetic susceptibility influence disease risk, and if this is mediated by the developing microbiome and epigenetic mechanisms. In addition, it is possible to undertake studies within subpopulations and to integrate population-based interventions within the design.

## Methods

### Aims and objectives

The overall aim is to build a large database and biobank, and investigate how exposures in early life influence health, development, and growth in childhood. Specific objectives are to identify early predictors in the development of early-onset NCDs including allergic diseases, overweight/obesity, cognitive and behavioral problems, gastrointestinal disorders, and caries in children until 7 years of age.

### Study design, setting and participants

**Fig. 1:**
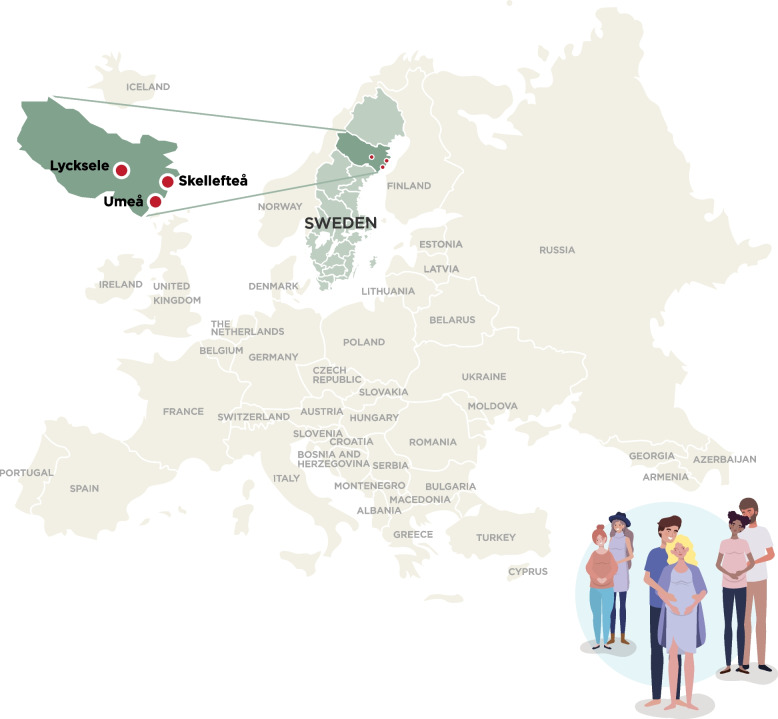
Study setting

This is an epidemiological prospective population-based longitudinal birth cohort study aiming to recruit 10,000 families i.e., 30,000 individuals (the pregnant woman, her partner, and their child). Västerbotten County is situated in northern Sweden, covers one eight of Sweden’s area and has more than 270,000 inhabitants with 80% residing in the coastal region [56]. There are ~ 3,000 deliveries yearly at three delivery wards situated at Umeå University Hospital, Skellefteå Hospital and Lycksele Hospital within Västerbotten County (Fig. 1). The study was registered at www.researchweb.org/is/en/sverige (project number 279272).

### Recruitment

All pregnant women in Västerbotten County are invited to participate in NorthPop together with their partner and a study brochure with information about the study is sent to the pregnant women two weeks prior to a routine ultrasound examination at 14 to 24 weeks' gestation (Fig. 1). Recruitment takes place at the time of the routine ultrasound examination at one out of three Special Maternity Health Care Units in Västerbotten county, and additional information about the study is given by trained NorthPop study staff. Both parents-to-be are required to provide informed consent for us to be allowed to study their child. If the partner is not present at the routine ultrasound examination, informed consent is collected after the appointment but before inclusion in the study. If the pregnant woman has no partner, she provides informed consent for her and her child. Recruitment started in 2016 at Umeå University Hospital (with the largest delivery unit); was expanded to include Skellefteå Hospital (with the second largest delivery unit) in 2019 and the county’s third delivery unit at Lycksele Hospital in 2021. More than 60% of the eligible population is currently included in the cohort. Recruitment is expected to end in 2025.

Inclusion criteria are pregnant women ≥ 18 years of age, comprehending the Swedish language, viable pregnancy at 14 to 24 weeks' gestation, and intention to give birth and reside in the catchment area for the next few years. The children and their parents are followed primarily until the child turns 7 years old. Both parents provide informed consent, which also includes the possibility to access data from other local and national databases such as the health care medical and dental records system, as well as nationwide registers (Table 1).

**Table 1:** External registers in the NorthPop database

Register name	Description of variables
Child health care records	Growth, nutrition, development, sight, hearing
Dental records	Oral health of child and parents
Maternity care center records	Assessments during pregnancy
School health care records	Growth, eyesight, hearing, well-being, referrals
The Longitudinal Integrated Database for Health Insurance and Labour Market Studies (LISA) [57]	Socioeconomics
The National Patient Register [58]	Data on health care episodes in inpatient and outpatient specialist care
The National Prescribed Drug Register [59]	Prescribed drugs
The Swedish Child Health Services Register (BHVQ) [60]	Growth, nutrition, development, sight, hearing
The Swedish Geographical Database [61]	Geographic data
The Swedish Neonatal Quality Register (SNQ) [62]	Neonatal intensive care
The Swedish Pregnancy Register [63]	Pregnancy-, childbirth- and neonatal outcomes
The Swedish Quality Register for Caries and Periodontitis (SKaPa) [64]	Oral health of child and parents
The Swedish School Health Care Quality Register (EMQ) [65]	Growth, sight, hearing, well-being, referrals
The Total Population Register [66]	Marital status, family relationships and migration

### Data collection

**Fig. 2:**
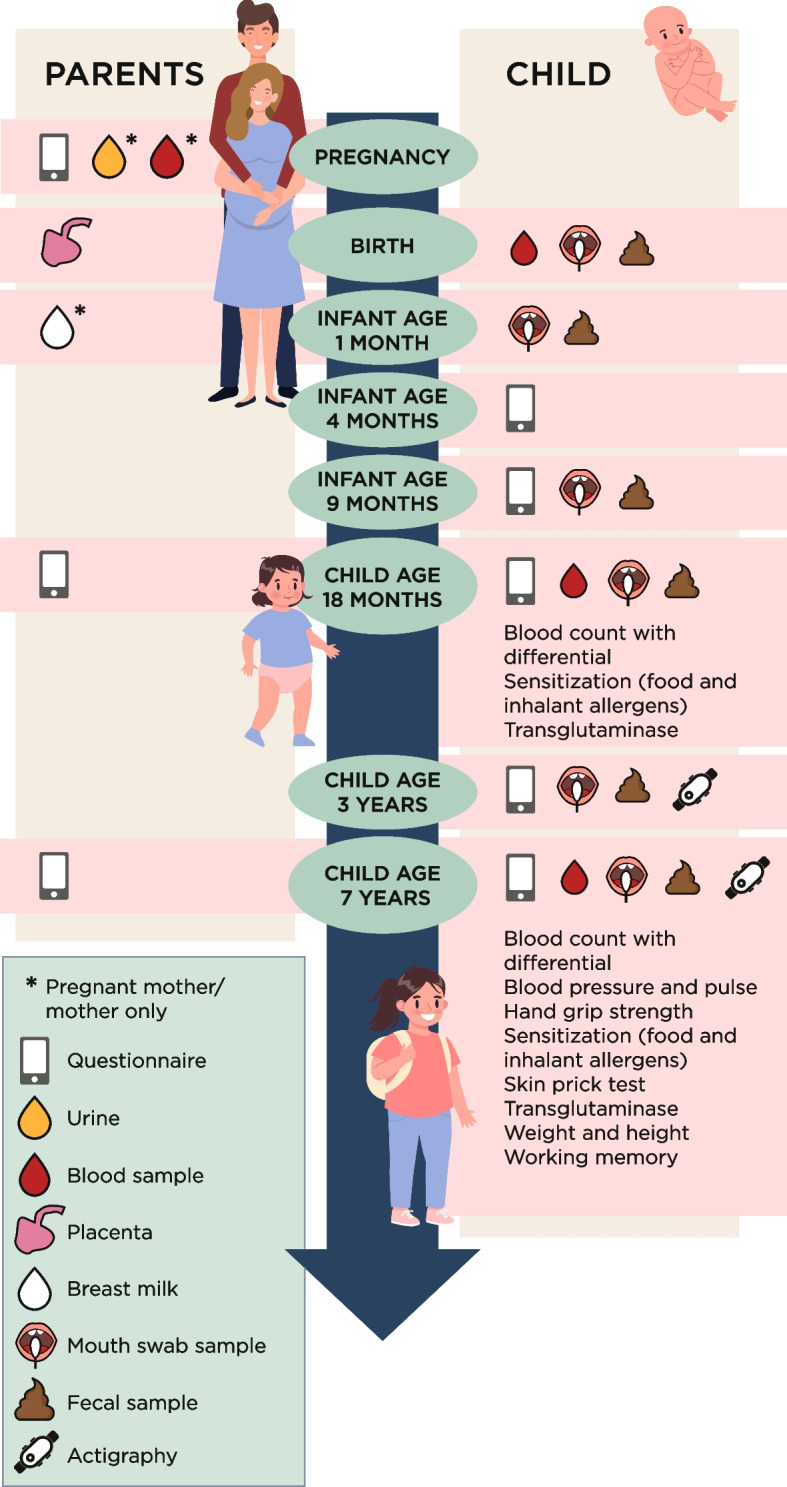
Overview of data collection and samplings

The data collection includes web-based questionnaires with items on parental, fetal and child health outcomes, lifestyle, diet, and environmental exposures starting in pregnancy and continues in childhood until the children turn 7 years old (Tables 2 and 3, Fig. 2). As displayed in Tables 2 and 3, validated as well as newly constructed questionnaires are applied. An advanced survey software platform is utilized to administer questionnaires to the participants through automated text and email messages. During pregnancy, four questionnaires are to be completed by the pregnant woman and four by the partner. A series of web-based questionnaires are administered when the child is 4 months, 9 months, 18 months, 2 years, 3 years, and 7 years old (Tables 2 and 3). Some of the questionnaires are divided into parts and are sent in intervals of weeks or months.

**Table 2:** Questionnaires relating to parental health and lifestyle

	Gestational age 14–24 weeks	Gestational age 26 weeks	Gestational age 34 weeks	Infant age 4 months	Infant age 9 months	Child age 18 months	Child age 2 years	Child age 3 years	Child age 7 years
Mother*	Country of birth	Cravings	Diet	Medications during pregnancy	Hand sanitizer use	Parental leave	Diet	Mental health ^a^	Diet
	Heredity	Exposure to diseases and infections	Exposure to UV radiation	Physical and mental health	Parental leave		Physical activity	Taste preferences	Physical activity
	Housing and home environment	Mental health ^a^	Physical activity	Pregnancy complications	Tobacco use		Physical and mental health ^b^		Physical and mental health ^c^
	Physical and mental health	Nausea and vomiting	Sick leave	Social media use			Socioeconomics		Socioeconomics
	Socioeconomics	Pregnancy complications and medication	Travel abroad	Weight					Tobacco use
	Weight and height	Tobacco and drug use	Vaccination						Weight and height
Co-parent*	Country of birth	Exposure to diseases and infections	Diet		Hand sanitizer use	Parental leave	Diet		Diet
	Heredity	Mental health ^a^	Physical activity		Parental leave		Physical activity		Physical activity
	Housing and home environment	Tobacco and drug use			Tobacco use		Physical and mental health ^b^		Physical and mental health ^c^
	Physical and mental health						Socioeconomics		Socioeconomics
	Socioeconomics								Tobacco use
	Weight and height								Weight and height

^a^Including the General Health Questionnaire (GHQ-12) [67]

^b^Including the General Health Questionnaire (GHQ-12), Social support scale [68], Mindful Attention Awareness Scale (MAAS-5) [69], Perceived Stress Scale (PSS-10) [70]

^c^Including the General Health Questionnaire (GHQ-12) and the Mindful Attention Awareness Scale (MAAS-5)

^*﻿^A corona-specific questionnaire including the COVID-19 Peritraumatic Distress Index (CDPI) was administered to all participants at four time points between 2020 and 2021 with questions for parents and child [71]

**Table 3:** Questionnaires relating to child health and exposures

Infant age 4 months	Infant age 9 months	Child age 18 months	Child age 3 years	Child age 7 years
Antibiotics	Antibiotics	Antibiotics	Antibiotics	Allergic rhinitis
Diet	Asthma	Asthma	Asthma	Antibiotics
Eczema ^a^	Baby swimming	Breastfeeding	Bedtime routine	Asthma ^g^
Exposure to chemicals	Diet	Child care	Child care	Bedtime routine
Feeding problems	Eczema ^a^	Diet	Diet	Diet
Housing and home environment	Food allergy	Digital devices	Digital devices	Digital devices and social media
Neurodevelopment ^b^	Functional gastrointestinal disorders ^c^	Eating behaviour and feeding problems ^d^	Eating behaviour and feeding problems ^d^	Eating behaviour and feeding problems ^h^
Neonatal intensive care unit admission	Housing and home environment	Eczema ^a^	Eczema ^a^	Eczema ^a^, ^g^
Pacifier use	Infections	Food allergy	Emotional and behavioral difficulties ^e^	Emotional and behavioral difficulties ^i^
Sleeping habits and crying	Neurodevelopment ^b^	Hospitalization	Finger-sucking	Eyesight
Temperament	Pacifier use	Housing and home environment	Food allergy	Food allergy
Time spent outdoors	Teething and oral health	Infections	Handedness	Housing and home environment
	Wheezing	Neurodevelopment ^b^	Housing and home environment	Infections
		Pacifier use	Infections	Neurodevelopment ^j^
		Sleeping habits	Neurodevelopment ^b^	Oral health
		Teething and oral health	Oral health	Physical activity ^k^
		Temperament	Pacifier use	Reading
		Time spent outdoors	Physical activity ^f^	School environment
		Vaccines	Sleeping habits	Sleeping habits
		Wheezing	Temperament	Taste preferences
			Time spent outdoors	Time spent outdoors
			Wheezing	Wheezing ^h^

^a^Including the Patient Oriented Eczema Measure (POEM) [72]

^b^Including the Ages & Stages Questionnaires®, Third Edition (ASQ®−3) [73]

^c^Including the Rome IV Diagnostic Criteria [74]

^d^Including the Child Eating Behaviour Questionnaire (CEBQ) [75] and the Montreal Children’s Hospital Feeding Scale (MCHFS) [76]

^e^Including the Strengths and Difficulties Questionnaire (SDQ) [77]

^f^Including Early Years Physical Activity Questionnaire (EY-PAQ) [78]

^g^Including questionnaires from The International Study of Asthma and Allergies in Childhood (ISAAC) [79]

^h^Including the Child Eating Behaviour Questionnaire (CEBQ), the Montreal Children’s Hospital Feeding Scale (MCHFS), Avodiant/Restrictive Food Intake Disorder Brief Screener (ARFID-BS) [80] and the Pica, ARFID and Rumination Disorder ARFID Questionnaire (PARDI-AR-Q) [81]

^i^Including the Strengths and Difficulties Questionnaire (SDQ), The Swanson Nolan and Pelham rating scale for the diagnosis of the attention deficit disorder (SNAP IV) [82] and the Autism Spectrum Quotient 10 (AQ-10) [83]

^j^Including the Ages & Stages Questionnaires®, Research Edition (ASQ®−3), personal communication

^k^Including modified Early Years Physical Activity Questionnaire (EY-PAQ)

At 18 months of age, children are invited to give a blood sample. At 3 and 7 years of age, the children are invited for actigraphy assessment during which they wear an accelerometer with an integrated light sensor (MotionWatch 8, CamNtech, Cambridge, UK) 24 h per day for eight days, including a semi-standardized diary for the parents to complete. At 7 years of age, children are invited to a follow-up visit, including measurements of weight/height, blood pressure, hand grip strength, working memory, as well as collection of blood and saliva samples and a skin prick test (SPT). Hand grip strength for both hands is assessed using a Jamar PLUS + digital dynamometer (SN 2021111169) while the child is seated, with the upper arm positioned alongside the body and the elbow flexed at 90 degrees. Working memory is assessed using the Corsi block tapping task, including a backward task, adapted from an open-source cognitive test battery [84]. The SPT is administered on the volar forearm and include allergens Dermatophagoides pteronyssinus Derp1 and Dermatophagoides farinae, birch, timothy, dog, and cat (Soluprick SQ, ALK, Horsholm, Denmark). A test is considered positive if a wheal ≥ 3 mm is observed in response to any of the allergens in the presence of an appropriate response to the positive control (10 mg/mL histamine dihydrochloride; ALK, Horsholm, Denmark) and no response to the negative control (allergen diluents; ALK, Horsholm, Denmark).

### Collection of biological samples

Biological samples are collected from the woman during pregnancy, at gestational age 14 to 24 weeks, around gestational age 28 weeks, at delivery and 1 month postpartum. The children are sampled at birth, within 48 h postpartum, at 1, 9, 18 months as well as 3 and 7 years of age (Fig. 2). Meconium, feces, oral swabs and breast milk are sampled either by the parents at home or at the hospital (i.e., samples collected within 48 h postpartum). At the Child Health Care Units and at the hospital, pre-packaged sampling kits are available for parents to collect. Parents are requested to label all the samples with their child’s personal identity number together with date and time of sampling. They also fill in the same information on a delivery note that is packed together with the sample. The sample and delivery note are placed in a ziplock bag and put directly into the freezer at home until transport to the hospital or the Child Health Care Unit.

#### Urine

A 10 mL urine sample is collected from the mothers at gestational weeks 14–24.

#### Placenta

The placenta is collected after delivery, in a sterile sampling bag (Thermo Fisher Scientific, MA, US), and stored at −80 °C for later processing.

#### Blood

Venous blood samples from the women at around gestational week 28, at birth from the umbilical cord and from the children at 18 months and 7 years of age are drawn into 6 mL EDTA tubes (Becton Dickinson, Plymouth, UK). When sampling children aged 18 months, blood is collected in two separate EDTA tubes, 6 ml and 0.4 ml, and one 3.5 ml SST II advanced serum tube (both from Becton Dickinson, Plymouth, UK). When sampling children aged 7 years, blood is collected in two separate EDTA tubes, 6 mL and 0.4 mL (both from Becton Dickinson, Plymouth, UK).

#### Meconium

Meconium is sampled, when voided, in a sterile fecal collection container with a spoon attached to the screwcap (Sarstedt AG & Co. KG, Germany). Parents are asked to record on the delivery note if sample is taken from the first pass meconium or at a later occasion. The sample is labelled and immediately placed in the freezer at −20°C and then transported frozen for long term storage at −80°C in the Northern Sweden Biobank.

#### Oral swabs

Parents collect oral swabs from the children at 48 h postpartum, at 1, 9, 18 months and at 3 and 7 years of age. The saliva/mouth microbiota sample is taken using a sterile cotton swab (Applimed SA, Chatel-St-Denis, Switzerland). The buccal mucosa, tongue, and alveolar ridges are swabbed carefully. The cotton swab is inserted into an Eppendorf tube (Sarstedt, Nümbrecht, Germany) with 100 µl TE-buffer (10 mM Tris, 1 mM EDTA, pH 7.6), spun properly in the buffer and then picked up and discarded, this procedure is then repeated once with an additional swab which is inserted into the same Eppendorf tube. Parents are asked to close the lid properly, label the tube and put it directly into the freezer. At 48 h postpartum the sampling is done at the hospital and at all other occasions the sampling is done at home. Parents are asked to label the tube and place it in the freezer until the samples are transported frozen to the hospital or the child health care unit and thereafter transported for long term storage at −80°C in the Northern Sweden Biobank.

#### Feces

The parents collect fecal samples at home, at 1, 9, 18 months and at 3 and 7 years of age. A sterile fecal collection container with a spoon attached to the screwcap (Sarstedt AG & Co. KG, Germany) is used to pick up stool from the infant’s diaper. For older children a paper tray is used to first collect the stool, which is then transferred into a sterile container. Parents are asked to label the tube and place it in the freezer until the samples are transported frozen to the hospital or the Child Health Care Unit and thereafter transported for long term storage at −80°C in the Northern Sweden Biobank.

#### Breast milk

When the infant is 1 month old, breast milk is sampled at home at the first feeding in the morning. Mothers express up to 30 mL of breast milk. Breast milk is expressed either manually or with a breast milk pump, the method used is recorded on the delivery note together with the number of hours passed since the last time the infant was breastfed and if the mother, at the time of sampling, was under antibiotic treatment. Breast milk is collected into a sterile 50 mL tube, mixed by inversion, and then divided into three sterile 10 mL tubes. Mothers are asked to label the tubes and place them in the freezer until the samples are transported frozen to the hospital or the Child Health Care Unit and thereafter transported for long term storage at −80°C in the Northern Sweden Biobank.

#### Saliva

When the child is 7 years old, whole saliva is collected into sterile test tubes while chewing on a 1-g piece of paraffin wax. Fresh saliva is then immediately transferred and placed in −20°C freezer and stored until transport for long term storage at −80°C in the Northern Sweden Biobank.

### Transport and storage of samples

#### Urine

 Urine samples are transported in-house from the Special Maternity Health Care Unit, at room temperature, to the biobank twice daily. At the Northern Sweden Biobank, samples are registered, centrifuged, aliquoted and stored at −80°C.

#### Placenta

 After delivery the placentas are stored in a −80°C freezer at the delivery ward and then transferred frozen, by study staff, to −80°C freezers at the research laboratory facility where placental tissue is processed.

#### Blood

Blood samples at gestational week 28 are collected at the Antenatal Care Units. Samples are stored at 4 °C until transport and are then transported at 2–10°C to the hospital laboratory, either the same day or the following workday. All samples are transported to the Northern Sweden Biobank for processing and freezing. Samples arriving at the laboratory after working hours are stored at 4 °C overnight and sent to the Northern Sweden Biobank the next morning. Cord blood samples are handled and frozen at −80°C within a target window of 1–2 h of collection. Samples collected during working hours are sent to the biobank for processing and freezing whereas samples collected after working hours are handled and frozen at the Center for Laboratory Medicine and later moved deep-frozen in −80°C to the Northern Sweden Biobank. All 6 mL EDTA blood samples, including umbilical cord blood, are centrifuged and plasma, buffy coat and red blood cells separated and aliquoted into new tubes. All aliquots are stored in the Northern Sweden Biobank at −80 °C. The 0.4 mL EDTA sample drawn at 18 months and 7 years of age is sent to the hospital laboratory for analysis of complete blood count with differential. Serum drawn at 18 months of age is sent at room temperature directly to the Clinical Immunology Laboratory for analysis and is not stored in the Northern Sweden Biobank. Serum drawn at 7 years of age is stored in the Northern Sweden Biobank.

#### Meconium, fecal samples, oral swabs, and breast milk

: At the delivery ward, the study participants are given a freezer bag including an ice pack and material for future samplings. To prevent samples from thawing, the parents are requested to use this bag to transport samples from home to the hospital or to the child health care unit where the samples are placed in a −20 °C freezer. Meconium samples are either placed in a −20 °C freezer at the delivery or maternity ward or, if sampled at home, parents are asked to bring the samples frozen (in the freezer bag) to the hospital at the time of the routine Newborn Blood Spot Screening, usually 48 h after delivery. Oral swab samples taken 48 h postpartum are placed in a −20 °C freezer at the maternity ward. Study staff collect samples from the wards every second week and bring them frozen to the Northern Sweden Biobank for registration and storage at −80°C. Study staff also routinely visit the Child Health Care Units and pick up samples for transport to the Northern Sweden Biobank. During transport, samples are kept at −20°C in a portable freezer. At the Northern Sweden Biobank, samples are registered and stored at −80°C.

#### Saliva 

Whole saliva is collected into sterile test tubes while chewing on a 1 g piece of paraffin wax. Fresh saliva is then immediately transferred to and placed in −20°C freezer and stored until transported for long term storage at −80°C in the Northern Sweden Biobank.

Studies with additional sampling are also done in sub-cohorts and examples of ongoing samplings and assessments are displayed in Table 4.

**Table 4:** Examples of planned and ongoing assessments

	Gestational age 14–24 weeks	Gestational age 28 weeks	Birth	Infant age 1 month	Infant age 9 months	Child age 18 months	Child age 3 years	Child age 7 years
Mother	Environmental pollutants Iodine	Environmental pollutants Genetics		Environmental pollutants Microbiomics				
Child			Environmental pollutants Epigenetics Genetics Microbiomics		Microbiomics	Blood count Environmental pollutants Metabolomics Microbiomics Proteomics Sensitization (food and inhalant allergens) Transglutaminase	Actigraphy Microbiomics	Actigraphy Blood count Blood pressure and pulse Environmental pollutants Hand grip strength Microbiomics Proteomics Sensitization (food and inhalant allergens) Skin prick test Transglutaminase Weight and height Working memory

### Data analysis, statistical methods, power calculation

Risk factors for health outcomes will be assessed using standard, hypothesis-driven methods, as well as data-driven methods. Power calculations for the NorthPop study have been performed with the aim to encompass many different exposures and outcomes. The targeted retention rate is 70%. Assuming a 30% loss of data due to missing values and attrition, power calculations are based on 7000 subjects in the whole cohort. According to Kooijman et al. [85], this will result in the following detectable relative risks (RR), with an alpha of 0.05 and a power of 80%: If the incidence of the outcome is 10% in the whole cohort, detectable RR will range from 1.55—1.23 as the proportion exposed varies from 5 to 50%. The corresponding detectable RR ranges are 1.80–1.33 and 3.09–1.83 if the incidence of the outcome is 5% and 1%, respectively. These power calculations are conservative since most studies will assess the effects of continuous instead of dichotomous exposures.

### Data storage and management

The main database is hosted by the Register Center North at Region Västerbotten with support from the Department of Information and Communication Technology Services and System Development (ITS) at Umeå University. The NorthPop data storage and collection are based on software by Forsta, London, United Kingdom. All servers are monitored by ITS using System Center Operations Manager (SCOM) and System Center Configuration Manager (SCCM). Backup is handled using Veeam Backup & Replication. Additional data are stored in a secure storage area hosted by Umeå University with a backup on an offline 256 bit advanced encryption standard (AES) encrypted hard disk kept in a locked room at the Department of Clinical Sciences, Umeå University. A full-time data manager is responsible for secure data handling including storage, backup, provision of access and processing for long-term storage. All data handling is done in accordance with the General Data Protection Regulation (GDPR). Metadata will be published at the Swedish National Data service (SND) repository within the project period. Long-term storage in generic formats for easy access also in the future is planned for all data in collaboration with Register Center North, Region Västerbotten and ITS, Umeå University.

### NorthPop consortium and access to data

NorthPop is currently in the recruitment and data collection phase but collected data are already available for researchers. Applications for use of NorthPop data are to be sent to the corresponding authors and reviewed by the steering group of the NorthPop research infrastructure.

## Discussion

This is a large, prospective, population-based birth cohort study with recruitment of pregnant women and their partners during the second trimester with follow-up of the children until at least 7 years of age. Our multidisciplinary, translational approach allows us to study how early life exposures, including diet, physical activity, daylight length, seasons, green environments, pollution, morbidities, and medication, as well as parental health and lifestyle, influence the programming of immune, metabolic, and neurodevelopmental pathways and future health in the offspring. We will also examine how these exposures interact with genetics, epigenetics and microbial colonization of the gastrointestinal tract, and influence future disease risk. We encourage multidisciplinary collaboration and the formation of new research interest groups within NorthPop. The steering group arranges yearly workshops that are open to all researchers. Examples of ongoing projects initiated by research interest groups include Allergy and immunity, Caries and oral health, Corona virus disease-19 (COVID-19), Digital media, Environmental pollutants, Epigenetics, Green environments and air pollution, Infections, Light exposure, Microbiome, Neurodevelopment, Nutrition, Physical activity, Sleep, Stress and 24 h- Movement Behavior.

Strengths include the extensive data collection of perinatal and early childhood environmental exposures using web-based questionnaires, biological samples from pregnant women and their children, and permission for linkage to medical records and national registers. The development of the research questions, outcome measures and study design have included involvement of parents. NorthPop researchers and staff have regular focus group discussions and structured telephone interviews with the participating parents on various aspects of the project including feedback during the development of questionnaires and data collection procedures. There are also possible limitations. Although our major clinical outcomes i.e., allergic diseases and asthma, overweight/obesity, cognitive and neurodevelopmental dysfunction, gastrointestinal disorders, and caries are prevalent in childhood, some analyses may be restricted by sample size and may need validation in other cohorts. Even though the participation rate is high, the extensive protocol and long follow-up may lead to selection bias and loss to follow-up.

## Conclusion

In this large population-based birth cohort study with recruitment in pregnancy we will investigate how early life exposures, as well as parental health and lifestyle, influence future health in the offspring. Our results are anticipated to contribute to the understanding of disease risk and may inform future strategies aimed at risk reduction, highly significant for individuals, public health and health sector policy.

## Data Availability

No datasets were generated or analysed during the current study.

## Abbreviations

NCDs: Non-communicable diseases
NorthPop: The NorthPop Birth Cohort Study
DOHaD: Developmental Origins of Health and Disease
PFD: Pediatric Feeding Disorder
ARFID: Avoidant Restrictive Food intake Disorder
ADHD: Attention Deficit Hyperactivity Disorder
SPT: Skin Prick Test
RR: Relative Risk
SCOM: System Center Operations Manager
SCCM: System Center Configuration Manager
AES: Advanced Encryption Standard
GDPR: General Data Protection Regulation
SND: Swedish National Data service
COVID-19: Coronavirus disease 19
